# The efficacy of conbercept or ranibizumab intravitreal injection combined with laser therapy for Coats’ disease

**DOI:** 10.1007/s00417-018-3949-1

**Published:** 2018-03-16

**Authors:** Longli Zhang, Yifeng Ke, Wei Wang, Xueying Shi, Kaiwen Hei, Xiaorong Li

**Affiliations:** 1 Vitreous and Retina Department, Tianjin Medical University Eye Hospital, Fukang Road 251, Nankai District, Tianjin, 300384 People’s Republic of China; 2 Vitreous and Retina Department, Hebei Eye Hospital, Xingtai, Hebei People’s Republic of China

**Keywords:** Ranibizumab, Conbercept, Coats’ disease, Intravitreal injection, Laser photocoagulation

## Abstract

**Purpose:**

The current treatment approaches for Coats’ disease by intravitreal injection of anti-vascular endothelial growth factor (VEGF) agents (ranibizumab or conbercept) combined with laser therapy were evaluated for the efficacy during the treatment.

**Methods:**

The medical records of 28 patients diagnosed with Coats’ disease followed by the treatment with intravitreal injection of anti-VEGF agents and laser therapies at Tianjin Medical University Eye Hospital and Hebei Eye Hospital during July 2012 and October 2017 were reviewed retrospectively. Clinical outcomes were recorded with a minimum follow-up of 6 months. The patients were divided into ranibizumab- and conbercept-treated groups, as well as based on age: pediatric and adult groups.

**Result:**

Twenty-eight patients were involved in this study. The average number of the injections was 2.82 ± 0.98. Laser photocoagulation was conducted in all patients, and the average number of lasers was 2.63 ± 0.74. The average follow-up period was 24.29 ± 9.85 months. Fourteen patients (50%) were stable, 12 (43%) patients were improved, and 2 patients (7%) showed recurred subretinal fluid and exudation. The final best corrected visual acuity (BCVA) increased markedly after intravitreal injection of ranibizumab or conbercept combined with laser therapy (*p* = 0.029, *p* = 0.009, respectively). The number of injections and lasers between conbercept and ranibizumab groups did not vary significantly (*p* = 0.160, *p* = 0.573, respectively). Nine patients (60%) in the ranibizumab-treated group and five (38%) in the conbercept-treated group reached a stable phase, and five (33%) and seven (54%) patients got the vision improved after treated with ranibizumab or conbercept, respectively. In pediatric and adult groups, the initial and final BCVA differed significantly (*p* = 0.03, *p* = 0.008, respectively). However, the injection number was remarkably different (*p* = 0.02), while the laser numbers did not have any markedly difference (*p* = 0.38).

**Conclusion:**

Intravitreal injection of ranibizumab or conbercept combined with laser therapy is an effective therapeutic option in Coats’ disease. Moreover, the intravitreal injection of ranibizumab or conbercept had no significant adverse effects and appeared to offer visual improvement in Coats’ disease.

## Introduction

Coats’ disease is an idiopathic progressive retinal vascular disorder, characterized by retinal telangiectasia and intraretinal and subretinal exudation that might lead to progressive exudative retinal detachment (RD) [1]. Coats’ disease is primarily observed unilaterally in early childhood, although some sporadic cases have been described in adults [2, 3]. The common signs include decreased visual acuity, strabismus, and leukocoria [4].

Shields et al. [1] classified Coats’ disease into five stages—stage 1, presence of retinal telangiectasia only; stage 2, telangiectasia and exudation (2A: extrafoveal exudation, 2B: foveal exudation); stage 3, exudative retinal detachment (RD) (3A: subtotal RD, 3B: total RD); stage 4, total RD and glaucoma; and stage 5, advanced end-stage disease.

The standard treatment involves ablation of the abnormal vasculature in order to achieve resolution of exudation and avoid disease progression. Laser photocoagulation and cryotherapy have conventionally been used for the treatment of early-stage Coats’ disease (stage 1 or 2). These techniques abolish both abnormal vessels and ischemic retina. On the other hand, the advanced stages of Coats’ disease (stages 3 and 4) are managed by various treatments including drainage of the subretinal fluid (SRF) and vitrectomy using gas or silicon oil [5]. Nevertheless, the management of Coats’ disease, especially advanced stage with RD, is yet challenging [6].

In recent years, intravitreal anti-vascular endothelial growth factor (VEGF) agents have been reported to reduce the SRF and exudation in Coats’ disease, both alone and in combination with conventional treatment modalities. However, a maximum number of studies continued to use bevacizumab, which is off-label and may have safety concerns [7, 8].

Currently, ranibizumab, a new anti-VEGF agent with humanized monoclonal antibody fragment targeting all isoforms of VEGF, applied in eyes with Coats’ disease has been reported. Nevertheless, the safety and efficacy data for ranibizumab in Coats’ disease is yet lacking, especially for children and the advanced Coats’ disease (stage 3 and over) [9, 10]. Conbercept is a recombinant fusion protein with a high affinity for all VEGF isoforms and has become a common therapy for the treatment of retinal diseases in China. However, the usage of conbercept in Coats’ disease has not yet been reported. Herein, we presented the treatment approaches for Coats’ disease by intravitreal injection of ranibizumab or conbercept in combination with laser therapy. Furthermore, we also evaluated the efficacy of ranibizumab and conbercept during the treatment.

## Materials and methods

The medical records of 28 patients, diagnosed with Coats’ disease followed by the treatment with intravitreal anti-VEGF agents (ranibizumab or conbercept) and laser therapy, at the Tianjin Medical University Eye Hospital and Hebei Eye Hospital, were reviewed during July 2012 and October 2017 retrospectively. This study was approved by the institutional review board of Tianjin Medical University and Hebei Eye Hospital, and the protocols adhered to the tenets of the Declaration of Helsinki.

The inclusion criteria for the patients were as follows. Firstly, patient with the stage 3 and over was involved. Idiopathic telangiectasia, aneurysms, and hard exudation involving at least one quadrant retina were confirmed. Secondly, patient presented treatment naivety before first visit and was treated with intravitreal anti-VEGF agents and laser photocoagulation. Subsequently, a minimum of 6-month follow-up was essential. Patients were excluded in the case of a known history of rhegmatogenous retinal detachment (RRD), intraocular inflammation, trauma, or any other diseases associated with retinal exudation.

The medical records including best corrected visual acuity (BCVA), logMAR (BCVA) = − log10 (BCVA), birth history, age, gender, family history, and medical history were collected, and systemic and other ocular abnormalities were assessed. These symptoms were confirmed during the first visit using fundoscopy, spectral domain optical coherence tomography (SD-OCT; OPKO Health, Inc., Miami, FL, USA), and fluorescein angiography (FA; Spectralis HRA, Heidelberg Engineering, Germany). A follow-up examination included BCVA, intraocular pressure, slit lamp examination, fundus photography, and SD-OCT. Some patients did not undergo all examinations due to the young age. Two investigators (Ke and Zhang) evaluated the results independently.

All patients were treated at the baseline using an intravitreal injection of ranibizumab (Lucentis; Genentech Inc., South San Francisco, CA, USA) or conbercept (Kanghong Inc., Chengdu, Sichuan, China). The alternative usage of the two agents was dependent on the financial situation of the patient. The reinjection was assessed after monthly follow-up. Intravitreal injection and laser therapy were performed under general or local anesthesia in the operating room. A dose of 0.5 mg/0.05 mL of ranibizumab or conbercept was injected into the vitreous using a 30-gauge needle. The pediatric and adult patients were using the same dose. Antibiotic eye drops (tobramycin eye drops; Alcon Laboratories Inc., Fort Worth, TX, USA) were applied to the injected eye four times daily 3 days before and after the injection.

### Groups

In order to compare the efficacy of two drugs, we divided the patients into ranibizumab-treated group and conbercept-treated group. Moreover, we also divided the patients into other groups based on the differences in age: pediatric and adult groups.

### Laser photocoagulation

In addition to the intravitreal injection, laser therapy was performed about 1 week after initial anti-VEGF injection. Laser photocoagulation was delivered in all areas of retinal telangiectasia in the case of little or no SRF. A 577-nm laser was used when local or general anesthesia was administered. The laser was done directly on the telangiectatic vessels. The clinical endpoint was the complete whitening of the telangiectatic vessels. The laser treatment in young patients, who did not cooperate, was delivered under general anesthesia at operation room. The average number of laser shots was 400 each time.

### Follow-up and outcome measurements

The follow-up was continued monthly after the injection. The additional injection was provided according to the follow-up fundus fluorescein angiography (FFA) or optical coherence tomography (OCT). When the patients showed new/increased SRF or sustained telangiectatic vessels, reinjection is recommended. New SRF is defined as recurrent exudative RD. Increased SRF is defined as the increased area of SRF or height of RD. Disease stability is defined as ≥ 3 visits without active leakage of fluorescein and macular exudation determined either by FFA or by OCT as well as regression of telangiectasia.

### Statistics

All statistical analyses were performed using SPSS 22.0 version statistical package (IBM Corporation, Armonk, NY, USA). All data are presented as the mean ± SD. The differences between the two groups (the conbercept and ranibizumab, the pediatric and adult) were assessed by *t* test. A two-tailed *P* value of < 0.05 was considered to be significant.

## Results

The cohort consisted of 28 patients. The baseline characteristics and treatment details are summarized in Table 1. The average number of the injections was 2.82 ± 0.98 (range one to five times). Laser photocoagulation was conducted in all patients, and the mean number of lasers was 2.63 ± 0.74 (range one to four times). The mean follow-up time was 24.29 ± 9.85 months. At the end of the follow-up, 14 patients (50%) were stable, 12 (43%) patients were improved, and 2 patients (7%) showed recurred SRF and exudation (patient 4, patient 6).

**Table 1:** The basic characteristics and treatment details in the 28 Coats’ disease patients

Patient no.	Age/gender/eye	Stage classification	Quadrants of telangiectasias	Initial BCVA	Final BCVA	Numbers of injection	Number of laser therapy	Follow up (months)	Termination	Retinal condition
1	4/F/os	3a	4	FC	20/200	Ranibizumab × 4	3	54	Improved	SRF, SHE
2	8/M/od	3a	2	20/200	20/133	Ranibizumab × 4	3	24	Improved	SRF, SHE
3	7/F/os	3b	4	HM	HM	Ranibizumab × 1	1	8	Worse	SRF, SHE
4	14/F/os	3a	3	20/100	20/50	Conbercept × 3	2	12	Improved	SRF, SHE
5	6/M/od	3a	4	HM	HM	Conbercept × 1	2	12	Worse	SRF, SHE, CME
6	5/M/os	3b	4	20/400	20/400	Ranibizumab × 5	3	20	Stable	SRF, SHE
7	5/F/os	3b	4	FC	FC	Ranibizumab × 3	3	15	Stable	SRF, SHE
8	6/M/od	3b	4	FC	FC	Conbercept × 3	3	36	stable	SRF, SHE, CME
9	5/M/os	3b	4	20/400	20/200	Conbercept × 3	3	28	Improved	SRF, SHE
10	8/F/os	3a	3	FC	20/200	Ranibizumab × 3	3	24	Improved	SRF, SHE
11	6/M/od	3a	4	FC	FC	Ranibizumab × 4	3	22	Stable	SRF, SHE, CME
12	5/M/od	3b	4	FC	FC	Conbercept × 3	3	36	Stable	SRF, SHE, CME
13	7/F/os	3a	4	FC	20/200	Ranibizumab × 4	4	28	Stable	SRF, SHE
14	10/M/od	3a	3	FC	FC	Ranibizumab × 4	3	24	Stable	SRF, SHE
15	6/M/od	3a	4	FC	FC	Ranibizumab × 3	3	32	Stable	SRF, SHE
16	8/M/os	3a	4	20/67	20/67	Ranibizumab × 3	2	28	Stable	SHE, CME
17	48/M/od	3a	3	20/100	20/50	Conbercept × 2	2	12	Improved	SHE, CME
18	42/F/od	3a	3	20/200	20/100	Conbercept × 2	2	17	Improved	SHE, CME
19	23/M/os	3a	4	HM	HM	Ranibizumab × 2	2	24	Stable	SRF, SHE
20	61/M/os	3a	2	20/1000	20/1000	Ranibizumab × 1	1	20	Stable	SHE, CME
21	60/M/os	3a	2	20/500	20/133	Conbercept × 2	2	11	Improved	SHE, CME
22	41/F/os	3b	4	20/200	20/100	Ranibizumab × 3	2	24	Improved	SHE, CME
23	33/M/od	3a	3	20/200	20/200	Conbercept × 3	3	28	Stable	SHE, CME
24	51/F/os	3a	3	20/200	20/200	Conbercept × 3	3	24	Stable	SRF, SHE, CME
25	34/M/od	3a	4	FC	20/400	Conbercept × 3	3	20	Improved	SHE, CME
26	49/M/od	3a	4	20/50	20/33	Conbercept × 2	3	38	Stable	SHE, CME
27	48/F/os	3a	4	20/200	20/100	Ranibizumab × 2	4	32	Improved	SHE, CME
28	39/M/os	3a	4	20/200	20/100	Conbercept × 3	3	27	Improved	SHE, CME

*BCVA*, best corrected vision acuity; *SRF*, subretinal fluid; *SHE*, subretinal hard exudative; *CME*, cystoid macular edema; *FC*, finger count; *HM*, hand motion

**Fig. 1:**
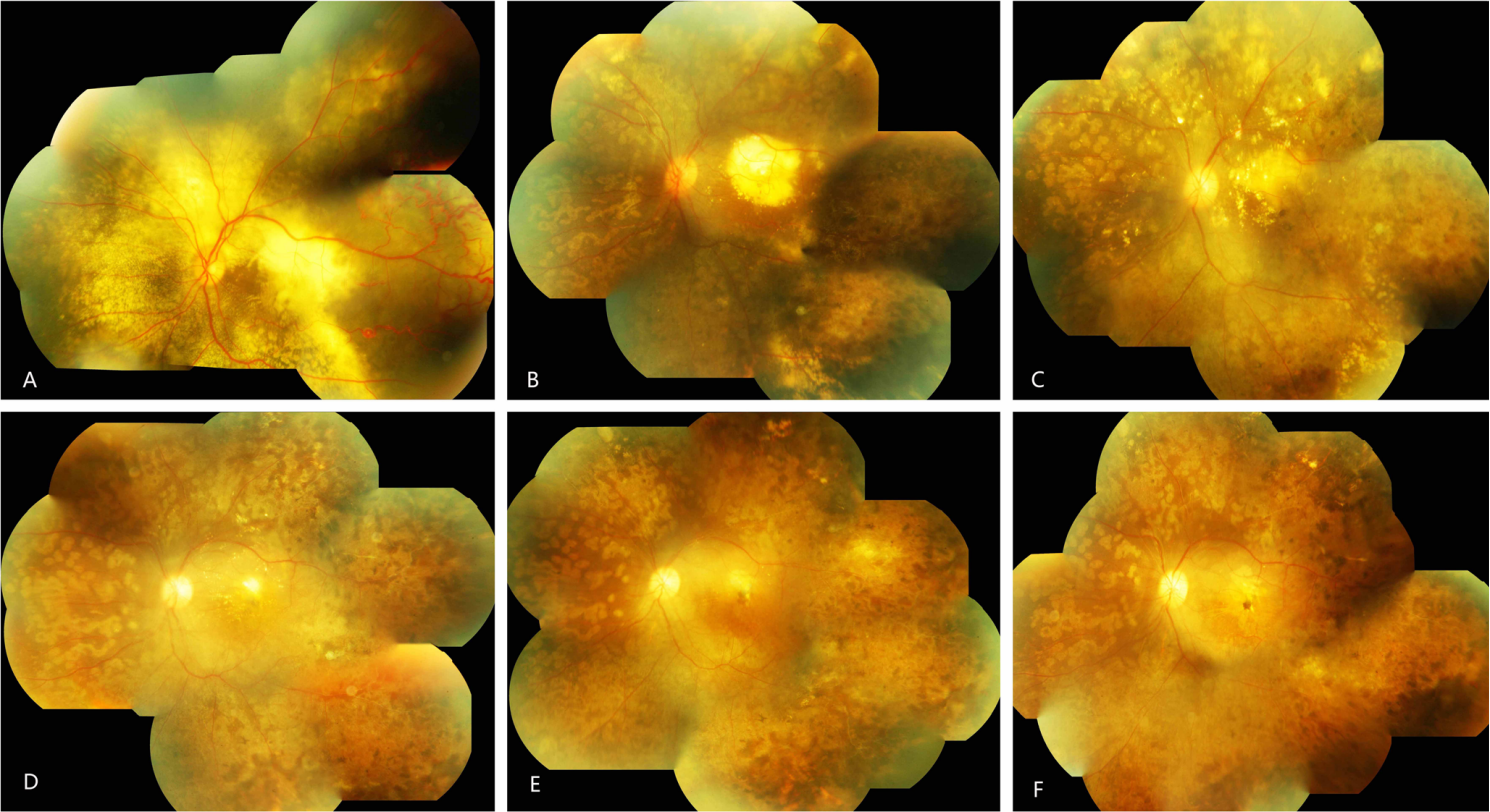
The fundus pictures of patient 1 from 2012 to 2017 (**a**–**f**). **a** The onset of Coats’ disease 2 months after initial one shot of laser and intravitreal injection. Telangiectasia and exudation in all quadrants of retina and exudative retinal detachment in posterior and peripheral retina. **b** One year after initial treatment. after three injections of ranibizumab and two times laser, the telangiectasia and exudative retinal detachment disappeared; exudation was limited around macular. **c** Eight months after **b**, Subretinal exudation was thinner and the macular exudation absorbed continuously. **d** One year after **c**, the subretinal and macular exudations were almost disappeared. **e** Thirteen months after **d**, the subretinal and macular exudations were completely absorbed. **f** One year after **e**, the macular had high-reflex scar at the end of follow-up. The lesion reached stable stage

**Fig. 2:**
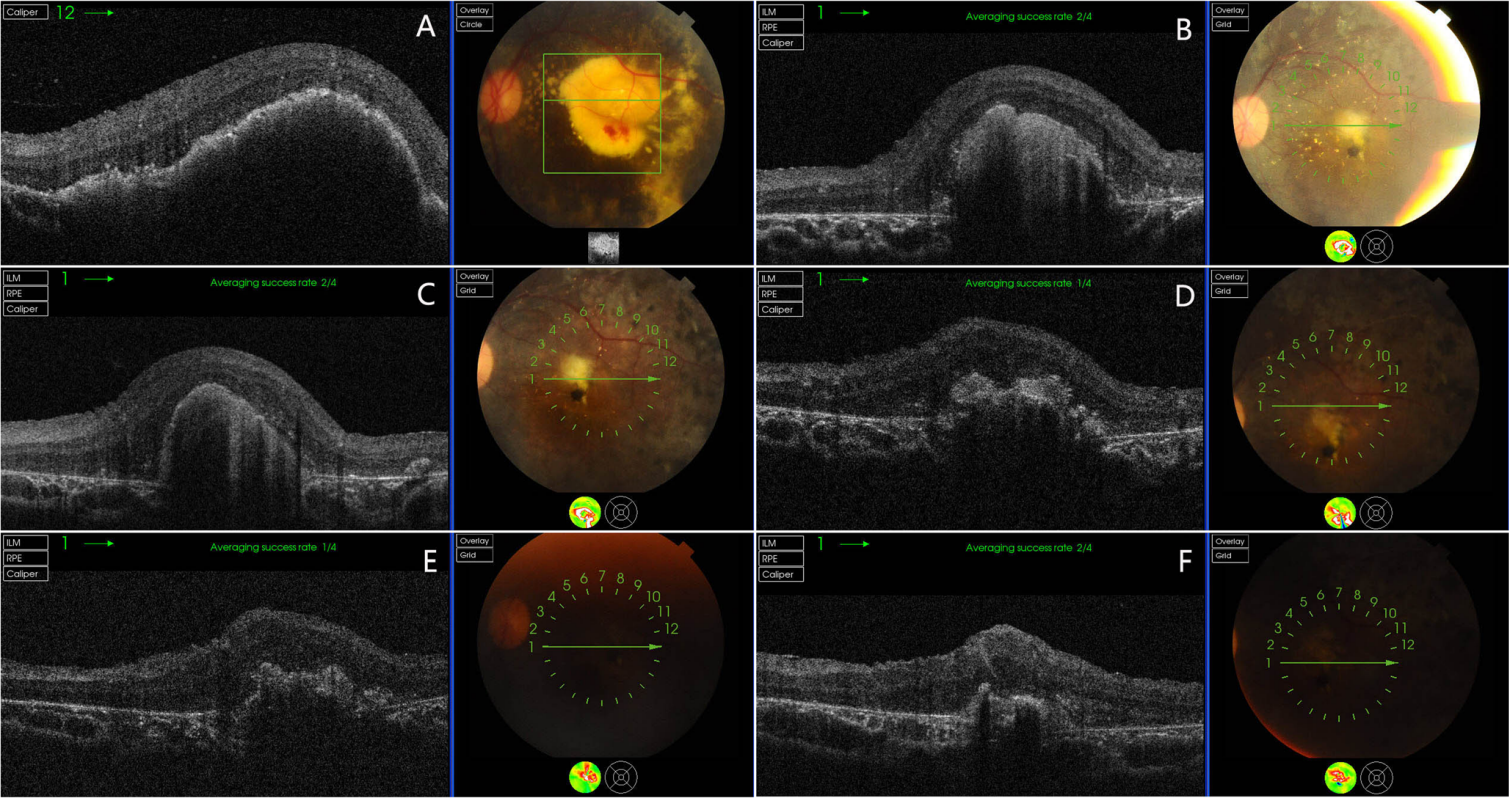
The OCT imaging of patient 1(**a**–**f**). **a** The macular exudation and edema at the onset 8 months after initial one laser and intravitreal injection. There was hemorrhage around the neovascularization. **b** One year after **a**, the macular exudation was limited in the center of macular. **c** Five months after **b**, the macular exudation absorbed slowly. **d** Six months after **c**, the macular exudation was absorbed continuously. **e** Seven months after **d**. **f** One year after **d**, macular exudation was completely disappeared and the subfoveal scar was confirmed

The logMAR BCVA was 1.57 ± 0.73 at baseline and 1.33 ± 0.81 at the final follow-up; the initial and final BCVA showed a remarkable difference after treatments (*p* = 0.000). A significant correlation was established between the baseline VA and the final follow-up VA (*r* = 0.96, *n* = 32), indicating that patients with better initial VA had relative good prognosis. All patients showed regression of the telangiectasia. Fifteen patients (88% of 17 SRF patients) demonstrated resolved SRF, and 15 patients (53% of 28 patients) had decreased subretinal hard exudation (SHE). After laser treatment, macular exudation in four patients (24% of 17 patients) had a complete resolution (Fig. 1). The cystoid macular edema decreased in 11 patients (73% of 15 patients) (Fig. 2). During follow-up, two children (patient 4, patient 6) exhibited unresolved recurrence of SRF and exudation. However, none of the patients experienced endophthalmitis, tractional retinal detachment, any other severe injection-related ocular, or a systemic adverse effect during follow-up.

For a continued comparison of the efficacy of ranibizumab and conbercept, the patients were divided into two groups (Table 2); 13 patients underwent intravitreal injection of conbercept and laser photocoagulation (conbercept group), and 15 patients were treated with intravitreal injection of ranibizumab and laser photocoagulation (ranibizumab group). Compared to the efficacy of both agents, our study demonstrated that the final BCVA increased significantly after intravitreal injection of ranibizumab and conbercept (*p* = 0.029, *p* = 0.009, respectively), indicating that the vision improved after anti-VEGF therapy and laser therapy. The number of injections and lasers between conbercept and ranibizumab groups did not vary significantly (*p* = 0.16, *p* = 0.573, respectively). Nine patients (60%) in the ranibizumab-treated group and five (38%) patients in the conbercept-treated group reached stable phase, and five (33%) and seven (54%) patients got the vision improved after treated with ranibizumab or conbercept, respectively, thereby suggesting a similar efficacy of both drugs with respect to stability.

**Table 2:** The comparison of ranibizumab and conbercept subgroups and pediatric and adult subgroups

	Patient No.	Initial BCVA (logMAR)	Final BCVA (logMAR)	P1	Injection No.	P2	Laser No.	P3	Follow-up time
Ranibizumab*	15	1.77 ± 0.70	1.52 ± 0.80	0.029	3.07 ± 1.16	0.160	2.60 ± 0.83	0.573	25.30 ± 10.00
Conbercept*	13	1.35 ± 0.72	1.14 ± 0.78	0.009	2.54 ± 0.66		2.77 ± 0.73		23.2 ± 9.92
Child (less than 18 years)^#^	16	1.80 ± 0.69	1.57 ± 0.80	0.030	3.19 ± 1.05	0.020	2.75 ± 0.83	0.380	25.20 ± 11.30
Adult (more than 18 years)^#^	12	1.27 ± 0.69	1.05 ± 0.73	0.008	2.33 ± 0.65		2.50 ± 0.80		23.10 ± 7.80
Total	28	1.57 ± 0.73	1.33 ± 0.81	0.000	2.82 ± 0.98		2.63 ± 0.74		24.29 ± 9.85

P1 is the t test between the initial and final BCVA. P2 is the t test of injection numbers between “*” subgroups. P3 is the t test of laser numbers between “#” subgroups

Among the pediatric and adult groups, all the pediatric patients exhibited SRF and SHE, 15 patients had telangiectatic vessels in the 4 quadrants of the retina, and 4 patients had cystoid macular edema (CME). In the adult group, 11 patients had CME and SHE, 2 patients had SRF, and 6 patients exhibited telangiectatic vessels in the 4 quadrants of the retina. This indicated SRF was prone to appear in pediatric patients, while CME was usually happened in adult patients. The final BCVA increased significantly in pediatric and adult groups after anti-VEGF and laser treatment (*p* = 0.03, *p* = 0.008, respectively). The injection numbers between each group showed significantly difference (*p* = 0.02), indicating that the pediatric patients required prolonged injection due to severe symptoms, while the number of lasers did not differ markedly between each group (*p* = 0.38).

## Discussion

Currently, standard guidelines for the treatment of Coats’ disease are absent. The main objective of various therapeutic options, including laser photocoagulation, cryotherapy, and SRF drainage surgery, is to obliterate the abnormal vessels and minimize exudation. In the event of massive retinal exudation or serous RD, laser treatment or cryotherapy is often ineffective; therefore, SRF drainage surgery becomes imperative. Nonetheless, the SRF drainage surgery presents high risk of complications, especially in children. Hence, advanced Coats’ disease (stage 3 and over) has a poor prognosis. The pathogenesis of Coats’ disease is yet unclear; however, previous studies have suggested that VEGF may be involved. VEGF is a naturally occurring protein that causes increased vascular permeability, endothelial cell migration, and proliferation [11]. Several studies have found elevated levels of VEGF in Coats’ disease, thereby indicating its putative role in the pathogenesis of Coats’ disease. The increased levels of VEGF are speculated to result in an increased vascular permeability and formation of peripheral telangiectasias [12, 13]. Recently, anti-VEGF therapy has been shown to exert a rapidly expanding role in the treatment of Coats’ disease [10, 14, 15]. The elevated VEGF levels in Coats’ disease decreased markedly after the injection of the anti-VEGF agent [16]. These results support the intravitreal application of anti-VEGF therapeutics for the treatment of Coats’ disease.

Based on previous literature reports, we selected intravitreal injection of anti-VEGF agents as an initial treatment [9, 10]. The treatment with the anti-VEGF agent, bevacizumab, was reported in a majority of Coats’ disease [7, 8], while the usage of ranibizumab in Coats’ disease was rarely reported. Yang et al. analyzed 17 young patients of Coats’ disease and found intravitreal ranibizumab combined with laser and cryotherapies was an effective and a safe treatment approach for Coats’ disease that may improve the visual acuity and reduce the subretinal fluid, exudates, and telangiectasia ([10]. Although the intravitreal injection of conbercept in Coats’ disease was not yet reported, some studies reported that conbercept is reliable in ocular disease. Zhang et al. studied 98 eyes in AMD or diabetic macular edema (DME) and revealed that repeated intravitreal injections with conbercept showed an excellent safety profile for retinal nerve fiber layer thickness, although short-term IOP changes were observed [17]. Sun et al. reported 60 patients with macular edema secondary to retinal vein occlusion; these patients were treated with conbercept and presented non-ocular adverse events that were related to the drug or the injection procedure [18]. Therefore, we thought conbercept and ranibizumab could be used in the treatment of Coats’ disease.

In order to conduct a group comparison, ranibizumab or conbercept was selected depending on the patients’ financial condition as conbercept was cost-effective than ranibizumab. According to the current study, the final BCVA was improved both the conbercept-treated group and ranibizumab-treated group. We also realized that the initial BCVA in the ranibizumab group and conbercept group were 1.77 ± 0.70 and 1.35 ± 0.72, respectively. This revealed that the ranibizumab group, containing more pediatric patients, had poorer vision than the conbercept group. In addition, the VA in the ranibizumab group may not be accurate due to the young age of patients. Hence, the evaluation of drugs exhibited a deviation as vision served as an indicator. Therefore, we also use the remission of symptoms and stable lesions as the secondary indicators for assessment. In our study, the patients with stable or improved vision acuity of Coats’ disease were 14 (93%) and 12 (92%) in ranibizumab-treated group and conbercept-treated group, respectively. The two drugs displayed similar efficacy in relieving the symptoms associated with Coats’ disease.

Comparing the prevalence of adult patients, Coats’ disease usually affects young children, who are unable to report the symptoms. Until the patients visit the hospital, the disease becomes severe. The pediatric Coats’ disease commonly demonstrates SRF, hard exudation, and a large number of telangiectatic vessels. Nevertheless, the adult patients commonly present macular edema, exudation, and localized telangiectatic vessels [10]. In our study, the Coats’ disease in adults was not as severe as that in pediatric patients. CME is the main symptom that impacts the VA in adult patients. In addition to reducing edema by targeting VEGF as a major mediator, anti-VEGF therapy might also enhance the treatment efficacy in adult Coats’ disease [19]. Therefore, adult patients with Coats’ disease may have a better prognosis compared to pediatric patients. Macular exudation is another symptom that affects the BCVA in Coats’ disease. Yang et al. reported that it was difficult for the macular exudates to be absorbed completely; the authors speculated that the total regression of macular exudates was difficult and that the exudates of most patients were predisposed to hyper-reflective scar [10]. According to our current study, four (25%) patients showed complete absorption of macular exudates (Fig. 1). However, the BCVA of these patients did not improve due to the hyper-reflective scar (Fig. 2). Thus, the macular exudates can be observed as completely absorbed after a prolonged follow-up, although it does not entail that the vision can be rescued. The lesion may finally end in fibrotic scar and poor prognosis, especially in pediatric patients.

Injection and laser numbers are other key events in the treatment of Coats’ disease. Previous studies revealed that monthly injections could rapidly stabilize the symptoms [9–11]. Therefore, we utilized the anti-VEGF intravitreal injection as the initial treatment, then maintained a monthly follow-up period, and finally decided a reinjection according to the disease progress in patients. In our study, the numbers of injection and laser between conbercept and ranibizumab groups did not show significant differences. However, the pediatric patients were administered more injections than the adult patients, indicating that pediatric patients exhibited severe symptoms and achieving the stable stage were rather challenging. During the follow-up period, two patients (patient 3, patient5) showed unresolved recurred SRF after injection of anti-VEGF therapy and laser photocoagulation. Their parents refused reinjection due to financial reasons. Therefore, 26 patients (93%) did not get worse after combined treatment.

The adverse effects of anti-VEGF treatment for Coats’ disease are yet controversial. Some studies reported that Coats’ disease treated with intravitreal bevacizumab in addition to standard therapy could develop into vitreoretinal fibrosis and lead to tractional RD [20], whereas, other reports have shown that anti-VEGF agents might be able to reduce the fibrosis in non-ocular disease [21]. Furthermore, some results also indicated that there was no fibrotic change after utilizing intravitreal anti-VEGF agents in Coats’ disease [7, 10]. In the current study, we did not observe vitreoretinal fibrosis or traction in any patients during the follow-up period. Moreover, it has been proposed that vitreoretinal fibrosis is a natural occurrence during the Coats’ disease [1]. Thus, it remains uncertain whether anti-VEGF agents can accelerate the process. Along with advancing technology, the ranibizumab and conbercept are new designed specifically for the ocular disease. In recent years, any vitreoretinal fibrosis case has not been reported after anti-VEGF treatment. During our study, no patients experienced endophthalmitis, tractional retinal detachment, any other severe injection-related ocular, or a systemic adverse effect. Therefore, intravitreal injection of ranibizumab and conbercept can be considered as no significant adverse effects for the treatment of Coats’ disease.

Nevertheless, the present study has several limitations. Firstly, the sample size is small. The limited number of patients in this study resulted in a standard deviation, especially in subgroups. Secondly, the study did not have a control group, which would have allowed the evaluation of the true benefit of anti-VEGF treatment. Finally, our study did not include a standard treatment protocol; the reinjection of anti-VEGF agents or additional laser photocoagulation was administered at the clinician’s discretion according to the patient’s condition. All these parameters would impact the conclusion. However, as a preliminary study, it presented the reliability of intravitreal injection of ranibizumab and conbercept in Coats’ disease.

In conclusion, intravitreal injection of ranibizumab or conbercept combined with laser therapy is an effective therapeutic option in Coats’ disease. Moreover, the intravitreal injection of ranibizumab or conbercept had no significant adverse effects and appeared to offer visual improvement in Coats’ disease.

### Funding/support

Yifeng Ke was supported by grants from the Natural Science Foundation of Tianjin (grant number: 16JCQNJC12700) and the National Natural Science Foundation of China (grant number: 81500745). Xiaorong Li was supported by grants from the Natural Science Foundation of Tianjin (grant number: 15JCZDJC34500) and the National Natural Science Foundation of China (grant number: 81670875).
